# Biocontrol Efficacy and Mechanism of Action of *Bacillus velezensis* L33a Against Postharvest Sweet Potato Black Rot

**DOI:** 10.3390/jof12070492

**Published:** 2026-07-03

**Authors:** Wei Jian, Yuanyuan Li, Yaqian Zhu, Qing Yao, Youcheng Qin, Haiying Liu, Jing Zhang, Guoyang Qiu, Qihang Gui, Zhengwu Zhao

**Affiliations:** Key Laboratory of Plant Environmental Adaptation Biology of Chongqing, College of Life Sciences, Chongqing Normal University, Chongqing 401331, China; yyl1205999@163.com (Y.L.); 17838099207@163.com (Y.Z.); y17170207@163.com (Q.Y.); qyc020919@163.com (Y.Q.); l1251239232@163.com (H.L.); zhangj040110@163.com (J.Z.); qiuguoyang12138@163.com (G.Q.); gqh1124@163.com (Q.G.)

**Keywords:** *Bacillus velezensis*, sweet potato black spot disease, biological control, volatile organic compounds, *Ceratocystis fimbriata*

## Abstract

Black spot disease caused by *Ceratocystis fimbriata* (*C. fimbriata*) is a severe postharvest disease of sweet potatoes. This study evaluated the biocontrol potential of *Bacillus velezensis* (*B. velezensis*) L33a against this pathogen. Confrontation assays showed that L33a inhibited mycelial growth by 82.83%. FDA/PI staining and scanning electron microscopy revealed that L33a disrupted cell membrane integrity and caused severe mycelial deformation. Co-culture experiments indicated that L33a altered the expression of key pathogenic genes in *C. fimbriata*. Volatile organic compounds (VOCs) from L33a inhibited the pathogen by 77.78%, outperforming cell-free supernatant (CFS). VOCs primarily suppressed spore germination, with phenylethanol (PEA) and octanoic acid achieving 100% inhibition. In planta tests on sweet potato tubers showed that both L33a culture and VOCs significantly reduced lesion expansion. Using qPCR analysis, we found that L33a activated defense-related genes in tissues around wounds, particularly those involved in the jasmonic acid (JA) signaling pathway. In summary, *B. velezensis* L33a effectively controls sweet potato black rot through multiple mechanisms: direct antifungal activity, inhibition of spore germination, modulation of pathogen gene expression, and induction of host defense responses. It represents a promising natural inhibitor for postharvest disease management.

## 1. Introduction

Sweet potato (*Ipomoea batatas* (L.) Lam.) is an important industrial raw material and food crop, rich in nutrients such as dietary fiber, vitamins, and minerals [1,2]; all parts are edible [3]. China is the world’s largest producer of sweet potatoes, accounting for about 65% of the world’s total output [4]. The market demand and consumption preference for fresh sweet potatoes have become increasingly prominent as dietary structures change. However, sweet potatoes are highly susceptible to pathogenic fungi during post-harvest storage and transportation. Among these diseases, black spot disease—caused by *Ceratocystis fimbriata* (*C. fimbriata*)—is one of the major constraints on sweet potato yield and quality [5]. Upon infection, black lesions develop on the tuber surface, bitter compounds are synthesized, and toxic secondary metabolites—such as ipomeamarone (sweet potato ketone)—accumulate, posing serious threats to food safety and causing substantial economic losses in agricultural production [6]. Currently, black spot disease management still relies predominantly on synthetic fungicides, including carbendazim and thiabendazole [7]. Nevertheless, the long-term abuse of chemical pesticides not only leads to the development of resistance in pathogenic fungi, but also causes excessive pesticide residues, environmental pollution, and endangers human health [8]. Therefore, developing environmentally friendly, efficient, and safe biological control strategies has become an important direction of current research.

In recent years, the application of microorganisms and their metabolites in controlling various plant diseases has received extensive attention, involving multiple microbial groups such as fungi, bacteria, and actinomycetes. Studies have found that the salicylic acid hydroxylase gene (*Thshy1*) in *Trichoderma harzianum* can catalyze the generation of low-toxicity metabolites of fusarium acid, thereby effectively controlling *Fusarium* [9]. *Bacillus proteinolytic* L181 inhibits the growth of citrus acid rot pathogen through its volatile organic compounds (VOCs) [10]. The actinomycetes *Streptomyces lydicus* 6G-OA-10 achieves up to 97.36% control over the guacanthracides *Colletotrichum gloeosporioides* by producing the key antifungal substance mupirosin [11]. *Streptomyces djakartensis* MEPS155, isolated from the rhizosphere soil of sweet potatoes, also inhibits sweet potato black spot disease caused by *C. fimbriata* [12]. Among numerous biocontrol microorganisms, members of the genus *Bacillus* have received particular attention for their ability to form stress-resistant spores, produce multiple antifungal metabolites, and adapt to various environmental conditions.

*Bacillus* spp. exhibit broad-spectrum antagonistic activity against a variety of plant pathogenic fungi. Strains such as *Bacillus velezensis* (*B. velezensis*)HE-23, *Bacillus* sp. LNXM12, and *Bacillus siamensis* NEAU-ZGX24 effectively inhibit *Botrytis cinerea*’s infection in tomato plants [13,14,15]; and *Bacillus subtilis* KLBMPGC81 inhibits the growth of *Magnaporthe oryzae* [16]. *Bacillus* spp. also performs well in controlling sweet potato black spot disease. *B. velezensis* ATC-AL, *Bacillus tequilensis* XK29, *Bacillus altitudinis* P32-3, and *B. velezensis* CMML21-47 can significantly inhibit the black rot caused by *C. fimbriata* [17,18,19,20].

Microbial antagonism arises from a variety of mechanisms, including the production of lytic enzymes (such as chitinase and β-1,3-glucanase), VOCs, antibiotic compounds (such as lipopeptides and polyketones), and competition for nutrients and space [21,22,23,24]. Among these, VOCs represent one of the key mediators of this antagonistic activity. VOCs can exert antimicrobial effects without direct contact with food surfaces—offering a safer mode of action—and have thus become a current research hotspot [25]. It should be noted that studies on the biological control of black spot disease in sweet potatoes remain scarce. Existing work is largely limited to physiological and biochemical analyses—such as phenotypic observation and enzyme activity assays—with minimal investigation into pathogen-related gene expression changes in the causal agent and transcriptional regulation of host defense-related genes in sweet potato. This gap hinders a comprehensive understanding of the mode of action of biocontrol agents.

Our laboratory previously isolated a strain of *B. velezensis* L33a from soil and confirmed its excellent control effect against tomato gray mold. Whole-genome sequencing revealed multiple biosynthetic gene clusters encoding antimicrobial substances [26]. Based on this, this study further evaluated the biocontrol effect of L33a on black spot disease in sweet potatoes, systematically explored its mechanism of action, and focused on analyzing its direct antifungal activity, the effect of VOCs, the impact on pathogenic genes of pathogenic fungi, and the induction of host defense responses.

## 2. Materials and Methods

### 2.1. Plant Materials, Experimental Design and Treatments

*B. velezensis* L33a was previously isolated from soil samples from an orchard in Haikou, Hainan Province, China, and stored in a −80 °C refrigerator for future use. Strain *C. fimbriata*, donated by Professor Li Ming of Chongqing Normal University, was cultured and preserved on potato dextrose agar (PDA) medium at 28 °C. The tested sweet potato (*Ipomoea batatas* cv. Pushu 32) was collected from a planting base in Shapingba District, Chongqing City. The tubers were selected to be uniform in size, free of mechanical damage and disease, washed, disinfected with 2% sodium hypochlorite solution for 2 min on the surface, rinsed three times with sterile water, and air-dried in a clean bench for later use. 2-Tridecanol (98%) was purchased from Aladdin Industrial Corporation (Shanghai, China). 2-Nonanone (99%), octanoic acid (GC standard, ≥99.5% purity by GC), and phenylethyl alcohol (reagent grade, 98%) were purchased from Macklin Biochemical Co., Ltd. (Shanghai, China). All compounds were commercially available analytical standards and were stored at room temperature according to the suppliers’ recommendations.

### 2.2. Determination of In Vitro Antifungal Activity

The inhibitory activity of strain L33a against the black spot pathogen was determined using the plate confrontation method [27]. Fresh mycelial plugs (5 mm in diameter) of the *C. fimbriata*, cultured on PDA for 5 days, were placed at the center of PDA plates (90 mm in diameter). Subsequently, 10 μL of the L33a suspension (1 × 10^8^ CFU/mL) was spotted on each side of the plug, 2.5 cm away from its edge. Plates inoculated with the pathogen only served as controls. Each treatment was replicated three times. Plates were incubated at 28 °C for 13 days. The colony diameters of both the control and treatment groups were measured when the colony on the control group plates reached their maximum area, and the inhibition rate was calculated. The inhibition rate (IR) was calculated using the formula: IR (%) = (C − T)/C × 100, where C and T represent the average colony diameters (in mm) of the control and treatment groups, respectively.

### 2.3. Fluorescein Diacetate (FDA) and Propidium Iodide (PI) Staining

The FDA/PI staining method was used to assess the effect of L33a on the cell membrane integrity of the black spot pathogen, following a previously published protocol with minor modifications [28]. Samples were prepared as described in Section 2.2. Briefly, sterilized toothpicks were used to collect spores and hyphae from the colony edge of the fungal culture plate; the collected material was then suspended in equal volumes of FDA (50 mg/L) and PI (20 mg/L) solutions and incubated at 25 °C in the dark for 10 min. After washing with sterile water to remove excess staining, samples were observed under an RX50 series upright fluorescence microscope (SOPTOP/Ningbo Sunny Instruments Co., Ltd., Yuyao, China). Each treatment included three biological replicates, and each biological replicate was assessed in triplicate.

### 2.4. Scanning Electron Microscopy (SEM) Observation

The mycelial morphological changes in the *C. fimbriata* treated with L33a were observed using an SEM [29]. The sample was prepared in accordance with Section 2.2. After culturing for 5 days, the black spot mycelial masses (5 mm in diameter) were cut from the edge of the plate using a sterilized scalpel. All samples were fixed at 4 °C with 2.5% glutaraldehyde for 12 h, washed with 0.9% NaCl, dehydrated through a graded ethanol series, and subjected to critical-point drying followed by gold sputter coating. Finally, mycelial morphology was examined using a Hitachi SU3500 scanning electron microscope (Hitachi, Tokyo, Japan).

### 2.5. Determination of the Antifungal Activity of VOCs and Cell-Free Supernatant (CFS)

The inhibitory effect of VOCs produced by L33a on the black spot pathogen was determined using the double-plate buckling method [30]. Single colonies of L33a were picked from Luria–Bertani (LB) plates that had been cultured for 24 h and inoculated into 100 mL of LB liquid medium. The plates were cultured at 150 rpm at 37 °C for 12 h, and 100 μL of the liquid was evenly spread on LB plates (55 mm in diameter). PDA plates (55 mm in diameter) inoculated with black spot disks (5 mm in diameter) were inverted onto LB plates inoculated with L33a, with LB plates inoculated with 100 μL of ddH_2_O as the control, sealed with a sealing film, and cultured at 28 °C for 8 days. Each treatment was repeated 6 times. Continuously photograph and record, and measure the colony area.

Strain L33a CFS was prepared and briefly modified according to the method of Zhang et al. [19]. In brief, L33a was shaken in LB liquid medium at 37 °C for 24 h, and the supernatant was taken after centrifugation at 12,000 rpm and sterilized by filtration through a 0.22 μm membrane. Add CFS before PDA solidifies to achieve final concentrations of 10%, 20%, 30%, 40%, 50% (*v*/*v*). The stock CFS was the original supernatant harvested after centrifugation, without further concentration. Use PDA plates without CFS as the control treatment. Inoculate black spot plates (5 mm in diameter) in the center of the Petri dishes and incubate all plates at 28 °C for 8 days. 6 replicates for each treatment. Take consecutive photos to record and measure the colony area.

### 2.6. VOCs Inhibitory Effect on Spore Germination

Observe the effect of VOCs produced by L33a on spore germination of the *C. fimbriata* by referring to Xing et al., with slight modifications [31]. An appropriate amount of LB solid medium was added to one side of an I-shaped divided Petri dish, and 100 μL of L33a fermentation broth cultured for 12 h was inoculated onto it. After incubation at 37 °C for 24 h, an appropriate amount of potato dextrose broth (PDB) was added to the other side of the dish, and 6 mL of *C. fimbriata* spore suspension (1 × 10^6^ spores/mL) was inoculated. The Petri dishes were sealed with Parafilm and incubated at 28 °C for 1, 2, 3, 4, and 5 h, respectively. Samples were collected from the PDB at each predetermined time point for microscopic examination, with no fewer than 200 spores assessed per time point. Spore germination was observed under a microscope—germination was defined as the emergence of a germ tube whose length exceeded half the spore’s diameter—and the germination rate was calculated accordingly. Each treatment included six biological replicates, and each biological replicate was subjected to three technical replicates. The spore germination rate (%) was calculated as follows: Spore germination rate (%) = (number of germinated spores/200) × 100.

### 2.7. Co-Culture of C. fimbriata with Different Concentrations of L33a Fermentation Broth

Under aseptic conditions, *C. fimbriata* that had been cultured at 28 °C and 200 rpm for 5 days was taken and filtered through a sterilized 400-mesh sieve to obtain mycelium. A precisely weighed 2.5 g sample of *C. fimbriata* was transferred into 100 mL of PDB medium supplemented with L33a fermentation broth at volume ratios of 0%, 3%, 6%, 9%, and 12% (*v*/*v*). The resulting mixture was then co-cultured for 24 h. The mycelium was collected by centrifugation at 4 °C and 10,000 rpm for 10 min, washed 3–4 times with PBS [32]. Immediately drop into liquid nitrogen for rapid freezing, then store in a −80 °C refrigerator for later use. The mycelium samples collected from the co-culture were subsequently used for total RNA extraction and qPCR analysis to assess the expression of virulence-related genes (as described in Section 2.9).

### 2.8. In Planta Biocontrol Effect Determination of L33a

Refer to the method of Zhang et al., and make some minor modifications [33]. Select sweet potato tubers of uniform size, free from disease and damage. Wash them with running water to remove surface dirt, soak them in 2% sodium hypochlorite for 5 min for disinfection, then wash them with sterile water to remove residual sodium hypochlorite, and transfer them to the ultra-clean workbench for natural drying. Cut the sweet potatoes into 2 cm thick slices with a sterile scalpel. Make 1 cm-deep incisions at the center of each slice with a sterile toothpick, then inoculate 10 μL of L33a bacterial suspension (1 × 10^8^ CFU/mL) into each incision. After the bacterial liquid is air-dried, 10 μL of black spot pathogen spore suspension (1 × 10^6^ spore/mL) is inoculated. The control group was treated with an equal volume of ddH_2_O instead of the L33a bacterial suspension. The treated tubers were stored at 28 °C and 85–90% relative humidity for 8 days, and lesion diameters were measured and photographed every 2 days. Each treatment consisted of three tubers, and all treatments were replicated six times. To assess defense-related gene expression in tuber tissue, samples were collected from within 1 cm of the wound site at 0, 6, 24, and 48 h post-inoculation, immediately flash-frozen in liquid nitrogen, and stored at −80 °C until further analysis.

### 2.9. Quantitative Real-Time (qPCR) Analysis

Total RNA was isolated from sweet potato root and *C. fimbriata* samples using the RNAprep Pure Plant Kit (Tiangen Biotech Co., Ltd., Beijing, China). cDNA was synthesized from the isolated RNA using the PrimeScript™ RT Reagent Kit (Takara Bio Inc., Shiga, Japan). Subsequently, qPCR was performed on the Bio-Rad CFX system (Bio-Rad Laboratories, Inc., Hercules, CA, USA) by using SYBR^®^ Premix Ex Taq™ (Tli RNaseH Plus) (Takara Bio Inc., Shiga, Japan), with cDNA as the template. *CfActin* (from the *C. fimbriata*) and *IbActin* (from sweet potato) were used as internal reference genes, and relative gene expression levels were calculated using the 2^−ΔΔCt^ method. Primer sequences, amplification conditions, and thermal cycling program are listed in Supplementary Tables S1–S3.

### 2.10. Determination of in Planta Antifungal Effects of VOCs

Based on the method described by Pan et al., with slight modifications, the inhibitory effect of VOCs on postharvest black spot disease in sweet potatoes was tested [34]. In brief, 200 μL of L33a bacterial suspension was evenly spread onto 90 mm diameter LB agar plates and incubated at 37 °C for 24 h. Sweet potato slices (approximately 2 cm thick) were prepared and inoculated with *C. fimbriata* at the central incision as described in Section 2.8. Three inoculated slices were placed in a large Petri dish (15 cm diameter × 2.4 cm height). The two L33a plates pre-cultured for 24 h were then uncovered, and their bottoms (with the bacterial lawn facing upward) were stacked together and placed inside the same large dish. Another large Petri dish of identical dimensions (15 cm diameter × 2.4 cm height) was inverted and placed on top as a lid, forming an enclosed incubation chamber with a total height of approximately 4.8 cm. The assembly was immediately sealed with Parafilm and co-incubated at 28 °C with 85–90% relative humidity for 16 days. Photograph and measure the spot diameters every 4 days. LB plates inoculated with the same amount of ddH_2_O were used as controls.

### 2.11. Antifungal Effects of Monomer Volatiles

Place a (2 × 3 cm) sterile filter paper in a Petri dish (56 mm in diameter), add 100 μL of the monomer volatile compound to the filter paper, and then turn a PDA plate inoculated with a black spot mycelial disk (5 mm in diameter) upside down over the plate with the added compound. The control group added the same amount of ddH_2_O to the filter paper [35]. Six replicates for each treatment. All plates were incubated at 28 °C, and the fungal growth area was calculated once the *C. fimbriata* in the control group had completely covered the agar surface (8 days).

### 2.12. Data Analysis

Statistical processing of experimental data was performed using the GraphPad Prism 5 platform. Data are presented as mean ± standard deviation (SD). For single-factor designs, ordinary one-way ANOVA was used; for two-factor designs, two-factor ANOVA was used. Subsequently, Tukey’s honest significant difference (HSD) test was used for multiple comparisons, with *p* ≤ 0.05 considered statistically significant.

## 3. Results

### 3.1. L33a Inhibits Mycelial Growth of C. fimbriata and Disrupts Cell Membrane Integrity

**Figure 1:**
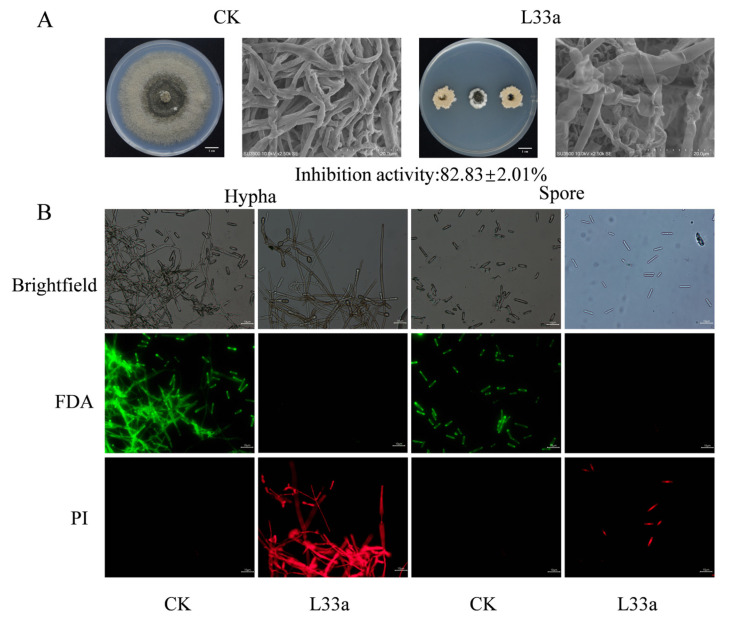
Anti-black spot activity of L33a in vitro. (**A**) L33a inhibitory effect on mycelial growth of *C. fimbriata* on PDA plate (bar = 1 cm) and SEM observation of the mycelium (bar = 20 μm). (**B**) FDA and PI staining of mycelium and spores of *C. fimbriata* (bar = 10 μm). The values represent the mean ± SD of the colony area from three media.

To evaluate the antagonistic activity of L33a against sweet potato *C. fimbriata*, two-point confrontation experiments were conducted. The results showed that, compared with CK, L33a had a significant inhibitory effect on the mycelial growth of the black spot pathogen, with an inhibition rate of 82.83%. SEM observations showed that the mycelium in the CK group was smooth and plump in shape, while the mycelium in the L33a treatment group showed severe twisting, wrinkling, collapse, and other abnormal shapes accompanied by mycelial swelling (Figure 1A). The FDA/PI staining results showed that the mycelium and spores in the control group were green fluorescence, indicating intact cell membranes; While some spores survived in the L33a treatment group, the hyphae showed red fluorescence, indicating impaired cell membrane integrity (Figure 1B). We hypothesized that L33a might have achieved its bacteriostatic effect by altering the cell membrane structure, making it difficult to maintain the mycelial structure.

### 3.2. L33a Affects Pathogenic Gene Expression in C. fimbriata

**Figure 2:**
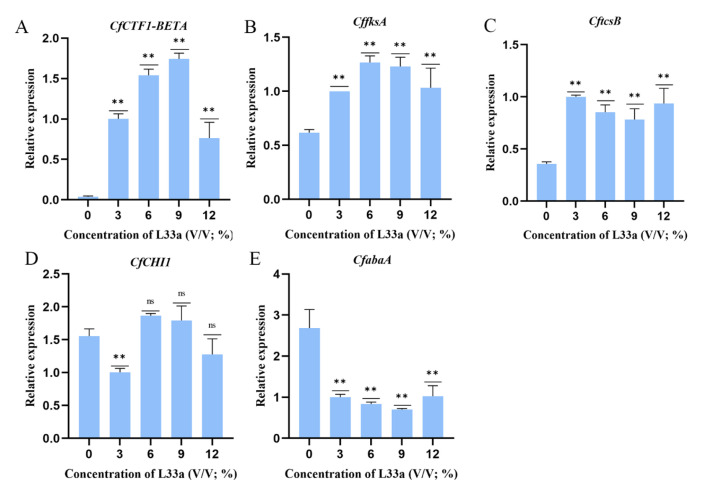
Effects of L33a on genes related to growth and pathogenicity of *C. fimbriata*. (**A**) *CfCTF1-BETA*. (**B**) *CffksA*. (**C**) *CftcsB*. (**D**) *CfCHI1*. (**E**) *CfabaA*. Data are expressed as mean ± SD. Statistical significance was evaluated using one-way ANOVA and Tukey’s HSD test, with ** *p* < 0.01 and ns indicating no significant difference.

qPCR analysis indicated that the expression of key genes associated with growth, virulence, and pathogenicity—including *CfCTF1-BETA* (a toxin secretion–related transporter), *CffksA* (β-1,3-glucan synthase involved in cell wall biosynthesis), *CftcsB* (transmembrane histidine kinase involved in osmosensing), *CfCHI* (chitinase involved in cell wall remodeling), and *CfabaA* (a central regulator of conidiation and infection-related morphogenesis)—was altered upon L33a treatment. As shown in Figure 2, under L33a treatment, the expression levels of *CfCTF1-BETA*, a gene that weakens the host barrier and inhibits the defense response, *CffksA*, a gene related to cell wall synthesis of *C. fimbriata*, and *CftcsB*, a gene related to infected plants, were all upregulated. The expression of the gene *CfabaA*, which is related to the regulation of *C. fimbriata* development, was significantly downregulated. In addition, *CfCHI*, a gene associated with cell wall degradation, was downregulated at 3% L33a and returned to the same level as CK when the concentration increased, but these changes were not correlated with the volume of L33a. This suggests that L33a treatment of biocontrol bacteria can regulate the expression of genes related to plant immunity, pathogen infection, and development, but this regulatory effect shows non-dose-dependent nonlinear characteristics.

### 3.3. The VOCs Produced by L33a Are the Main Active Antifungal Compounds

**Figure 3:**
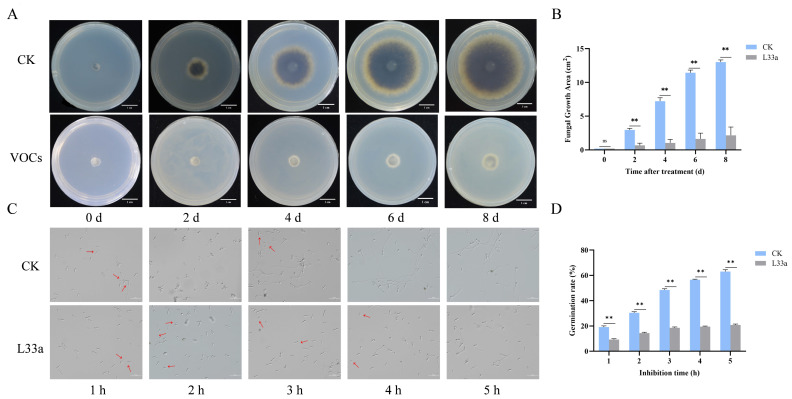
VOCs released by L33a inhibitory effect on *C. fimbriata*. (**A**) inhibitory effect on *C. fimbriata* (bar = 1 cm). (**B**) Effects on colony area of the *C. fimbriata*. (**C**) Effects on spore germination of *C. fimbriata* (bar = 50 μm). Red arrows indicate germinated spores. (**D**) Effects on spore germination rate. Data are expressed as mean ± SD. Statistical significance was evaluated using two-way ANOVA (factors: treatment and time point) followed by Tukey’s HSD test, ** *p* < 0.01 and ns indicating no significant difference.

To investigate the antifungal active substances of L33a, we conducted double-plate buckling experiments (VOCs) and fermentation filtrate (CFS) culture experiments, as shown in Figure 3A and Figure S1A. The results showed that both VOCs and CFS produced by L33a had inhibitory effects on the mycelial growth of the black spot pathogen. We recorded the area of plaque growth. As shown in Figure 3B and Figure S2B, plaque growth in the VOCs group was significantly inhibited compared with that in the CK group, and the inhibition rate reached 77.78% ± 2.48 on the eighth day of culture. Colony areas at the final CFS concentrations of 10%, 20%, 30%, 40%, and 50% (*v*/*v*) were 15.29, 11.02, 8.85, 6.92, and 4.43 cm^2^, respectively (Figure S1B). It was indicated that the inhibition rate gradually increased with the increase in CFS concentration. However, the inhibition effect of the VOCs treatment was better than that of the CFS treatment. Therefore, VOCs were used in the subsequent experiments. Microscopic observations revealed that VOCs had a significant inhibitory effect on spore germination (Figure 3C). After 5 h of culture, the spore germination rate of the control group was 63.02%, while that of the VOCs treatment group decreased to 20.82% (Figure 3D). The results suggest that VOCs may inhibit mycelial growth of the *C. fimbriata* by suppressing spore germination.

### 3.4. Analysis of the in Planta Control Efficacy of L33a Bacterial Suspension and VOCs Against Postharvest Sweet Potato Black Rot

**Figure 4:**
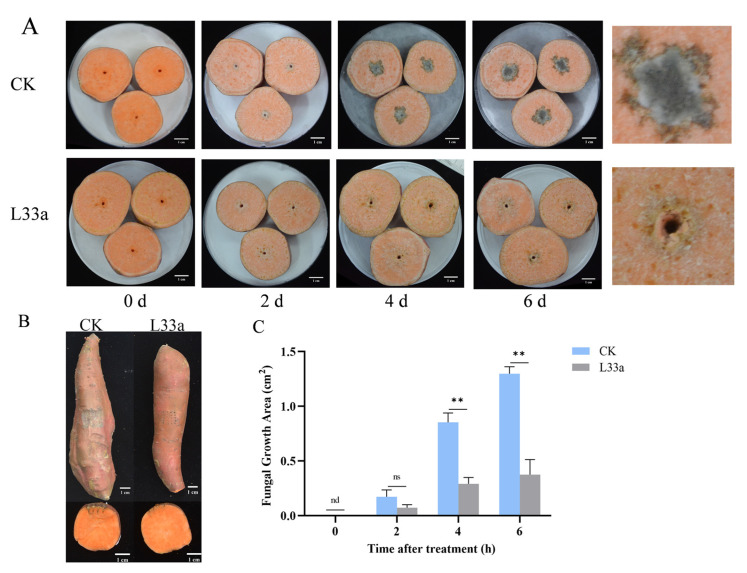
Biological control effect of *B. velezensis* L33a on postharvest black spot disease in sweet potatoes. (**A**) L33a inhibitory effect on postharvest black spot disease in sweet potatoes (bar = 1 cm). (**B**) Antifungal effect of L33a on postharvest intact sweet potatoes (bar = 1 cm). (**C**) Lesion area of the tuber. CK, blank control; L33a, L33a bacterial suspension treatment. Each group contained three sweet potatoes, and the experiment was repeated six times. All data are expressed as mean ± SD, ** *p* < 0.01, nd indicates no detection, ns indicates no significant difference.

In planta experiments showed that treatment with L33a suspension significantly inhibited lesion expansion in sweet potato tuber wounds. With increasing post-inoculation time, the lesion area in the CK group increased significantly, whereas the lesion in the L33a group—although expanding—was significantly suppressed and remained localized near the inoculation site (Figure 4A,B). Quantitative analysis of lesion areas using ImageJ (v1.52a, National Institutes of Health, Bethesda, MD, USA) revealed that the difference in lesion area between the CK and L33a groups became statistically significant starting from day 4 post-inoculation (Figure 4C). These results demonstrate that L33a exerts a strong antagonistic effect against *C. fimbriata* infection in sweet potato tubers under in planta conditions.

**Figure 5:**
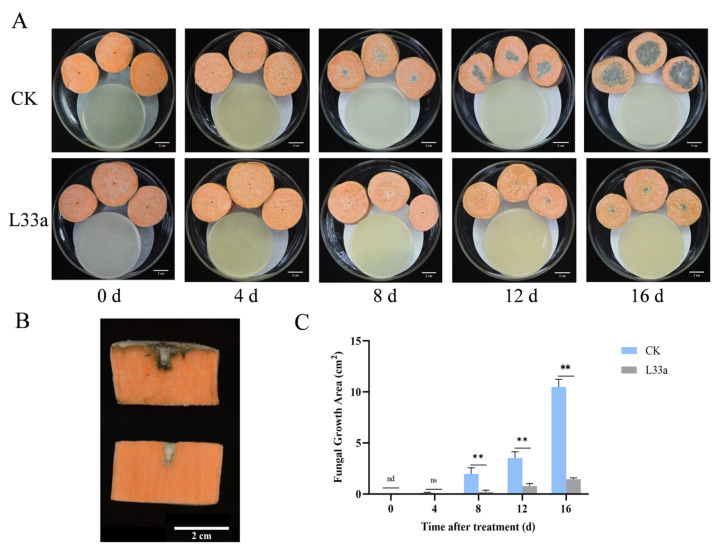
Biological control effect of VOCs produced by L33a on postharvest black spot disease in sweet potatoes. (**A**) Biological control effect of VOCs on postharvest black spot disease in sweet potatoes (bar = 2 cm). (**B**) *C. fimbriata* infection of sweet potato tubers transverse section (bar = 2 cm). (**C**) Area of the lesion. CK, blank control; L33a, VOCs fumigation treatment. Each group contained three tubers, data were the mean ± SD of six repeated trials, ** *p* < 0.01, nd indicated no detection, ns indicated no significant difference.

Likewise, the VOCs produced by the L33a strain were also highly effective in the biological control of postharvest black spot disease in sweet potatoes (Figure 5). As shown in Figure 5A, after 16 days of fumigation, the lesion area caused by the *C. fimbriata* on sweet potato tubers exposed to VOCs was significantly inhibited. When the sweet potatoes were cross-sectioned at the inoculation site, it was observed that pathogen growth was confined solely to the vicinity of the inoculation hole and did not spread to other parts of the tuber (Figure 5B). Quantitative analysis of lesion area revealed significant differences between the control (CK) and VOCs-treated groups starting from day 8 of incubation (Figure 5C). These results indicate that VOCs produced by L33a significantly suppressed colonization by the *C. fimbriata* in sweet potato tubers and delayed tuber rotting.

### 3.5. L33a Induces the Expression of Sweet Potato Defense Genes

**Figure 6:**
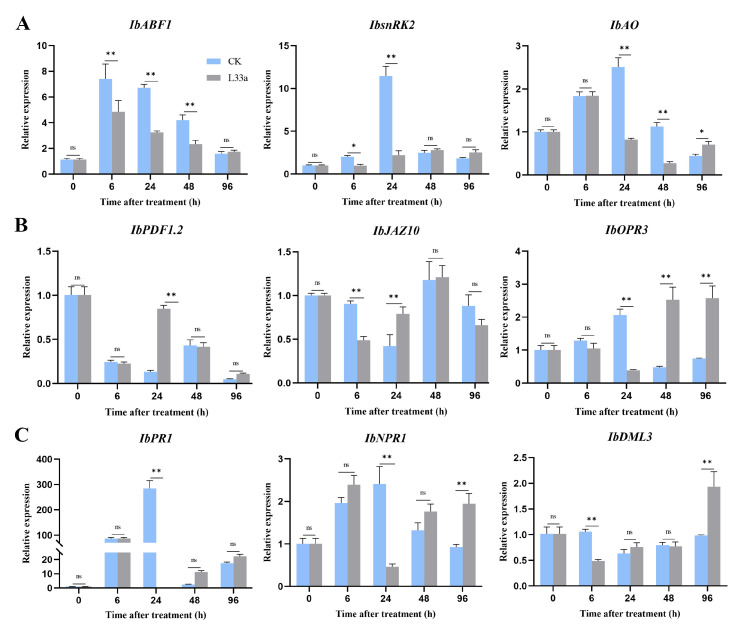
Detection of defense-related gene expression levels in sweet potato tubers treated with L33a inoculant. (**A**) ABA biosynthesis and signaling pathway. (**B**) JA biosynthesis and signaling pathway. (**C**) SA biosynthesis and signaling pathway. The values represent the mean ± SD of three independent samples, * *p* < 0.05, ** *p* < 0.01, ns indicated no significant difference. CK, blank control; L33a, L33a bacterial suspension treatment.

The expression of defense genes in sweet potato tubers was detected by qPCR to assess the potential of L33a to induce plant resistance. The analysis showed that most of the genes in the abscisic acid (ABA), jasmonic acid (JA), and salicylic acid (SA) pathways were induced by pathogen infection. Figure 6A shows that L33a can significantly inhibit the rapid expression of genes such as *IbABF1*, *IbSnRK2*, and *IbAO* in the ABA pathway in the early stage of infection (6–24 h), preventing their excessive activation and thereby reducing the stress damage caused by pathogenic bacteria; Figure 6B,C show that L33a continues to upregulate in the middle and late stages of the JA pathway marker genes *IbPDF1.2*, *IbJAZ10*, *IbOPR3* and the SA pathway core genes *IbNPR1*, *IbDML3*. At 24 h of treatment, the expression of *IbPDF1.2* and *IbJAZ10* increased by 6.46 and 1.87 times, respectively, but declined later. In addition, the expression levels of *IbAO*, *IbNPR1*, and *IbDML3* in the L33a treatment group were significantly higher than those in the control group at 96 h, increasing by 1.61, 2.09, and 1.97 times, respectively. More notably, the expression of *IbOPR3* after L33a treatment remained higher than that of CK after 48 h, reaching 5.25 and 3.46 times, respectively. Meanwhile, enzyme activation-related genes *IbPAL*, *IbCAT*, and *IbSOD* were significantly out expressed in the L33a treatment group in the later stage of treatment (Figure S2), with *IbCAT* reaching 34.715 times at 48 h (Figure S2C). In summary, L33a can control sweet potato pathogenic fungus by regulating the ABA pathway in the early stage and synergistically activating the JA-SA disease resistance cascade in the middle and late stages, presenting a sequential biphase biocontrol strategy.

### 3.6. Analysis of the Antifungal Effect of Monomer Volatiles

**Figure 7:**
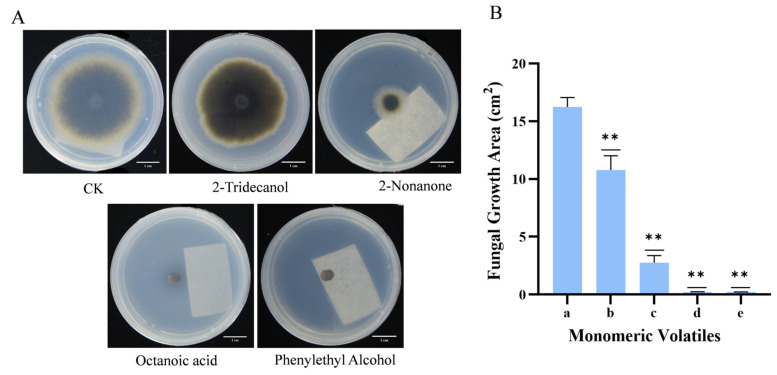
Inhibitory effects of four monomer compounds on *C. fimbriata*. (**A**) Inhibitory effect of four monomer compounds on *C. fimbriata* (bar = 1 cm). (**B**) Mycelial growth area. Note: a–e represent the fungal growth area under five different fumigation treatments. a: ddH_2_O; b: 2-tridecanol; c: 2-nonanone; d: octanoic acid; e: phenylethyl alcohol. CK, add an equal amount of ddH_2_O. Data are expressed as mean ± SD. Statistical significance was evaluated using one-way ANOVA and Tukey’s HSD test, ** *p* < 0.01.

Previous studies have identified the main components of VOCs in L33a [26]. To further identify the main VOCs that inhibit the *C. fimbriata*, considering safety, innovation, cost, and antifungal effect, four monomer volatiles, 2-tridecanol, phenylethyl alcohol (PEA), octanoic acid, and 2-nonanone, were screened. Each compound was used directly as purchased without dilution, and 100 μL of the pure compound was applied to a filter paper disk placed at the bottom of the Petri dish. The fungistatic effects were studied using the double-plate buckling method (Figure 7A). As shown in Figure 7B, 2-tridecanol, phenylethyl alcohol, 2-nonanone, and octanoic acid all showed significant inhibitory effects on *C. fimbriata*. Among them, PEA and octanoic acid showed particularly excellent inhibition rates against *C. fimbriata*, reaching 100%. After evaluating the safety of both, we further chose PEA to investigate its inhibitory effect on spore germination. Compared with CK, PEA fumigation significantly inhibited spore germination of *C. fimbriata* (Figure S3), which is consistent with the effect of VOCs fumigation of *C. fimbriata* spores produced by L33a in our previous study. It suggests that L33a is highly likely to suppress the occurrence of sweet potato black spot disease by generating the antifungal volatile PEA to inhibit spore germination.

## 4. Discussion

Sweet potatoes, as an important crop widely grown around the world, are widely used in food, feed, industrial raw materials, and other fields. However, sweet potatoes are vulnerable to sweet potato *C. fimbriata* during the seedbed, harvest, and storage periods, causing huge economic losses. This study found through confrontation experiments that L33a has a significant antagonistic effect on the black spot pathogen, with an inhibitory effect of up to 82.83 ± 2.01% (Figure 1A), which is consistent with existing reports on the inhibition of *C. fimbriata* by *Bacillus*. Unlike previous reports that mostly focused on the inhibitory phenotype and enzyme activity level, we investigated the biocontrol mode of action at the molecular level. On the one hand, qPCR confirmed that L33a could alter the expression of the pathogenic gene of *C. fimbriata*. On the other hand, L33a treatment induced temporal reprogramming of defense-related genes rather than simple sustained activation. Changes in JA pathway genes were particularly prominent. Compared with inoculation with the black spot pathogen alone, co-inoculation with L33a transiently activated the defense marker gene *IbPDF1.2* and the negative feedback regulator *IbJAZ10* at the early stage, followed by a rapid decline (Figure 6B). This pulse-like expression suggests that L33a triggers a fine-tuned negative feedback loop that delivers an early warning signal while avoiding excessive defense costs. Correspondingly, *IbOPR3*, a key rate-limiting enzyme gene in JA biosynthesis, was continuously upregulated at the mid-to-late stages, potentially driving sustained endogenous JA synthesis and laying the metabolic foundation for prolonged defense metabolism. The temporal uncoupling of early signaling initiation and later metabolic reinforcement indicates that the beneficial effect of L33a lies in orchestrating host defense into a perceive-first, fortify-later temporal strategy, rather than merely elevating the expression intensity of the pathway. Given that transcriptional changes in sweet potato under *Bacillus* treatment have been rarely documented, these findings reveal that L33a operates through a dual mode of pathogen suppression and resistance induction, providing a theoretical basis for the green prevention and control of postharvest black spot disease in sweet potato.

Fungal cell walls are rich in polysaccharides, which are crucial for maintaining the structure of fungal spores and the morphology of mycelium. Mycelium is the main form during fungal infection and colonization, and many studies have shown that damage to mycelium morphology may lead to a decline in its infectivity [36,37]. SEM analysis showed that the mycelium of the *C. fimbriata* treated with L33a was filamentous, deformed, folded, collapsed, and severely twisted (Figure 1B). After FDA/PI staining, it was found that the L33A-treated mycelium and spores emitted red fluorescence in the red fluorescence field and almost no luminescence in the green fluorescence field compared with CK, further indicating that the cell membrane structure was damaged.

The successful infection of the plant pathogen fungus depends on its precise regulation of a series of pathogenic genes related to growth, and development, cell wall degradation, signal transduction and metabolic adaptation. The study found that after L33a treatment, several key pathogene-related genes in the *C. fimbriata* were significantly upregulated (Figure 2), including *CfCTF1-BETA*, *CffksA*, *CftcsB,* and *CfCHI*, while the central developmental regulator *CfabaA* showed a downregulated trend. Studies have shown that the *Ctf1β* family of transcription factors are involved in fatty acid metabolism, cell wall stress response and infection regulation in fungi [38]; *FksA* is the encoding gene of β-1, 3-glucan synthase and is directly involved in the synthesis of the cell wall core skeleton [39]; TcsB, as a transmembrane heterozygous histidine kinase, plays a significant role in osmotic sensing and HOG MAPK pathways [40]; Chitinase (CHI) plays a dual role in fungal cell wall remodeling and stress adaptation [41]. The simultaneous upregulation of these four genes in this study may reflect the stress repair response initiated by the *C. fimbriata* under L33a stress, attempting to respond to damage by enhancing cell wall synthesis, sensing environmental changes, and remodeling the cell wall. However, this compensatory upregulation did not salvage its pathogenicity, and the root cause may lie in the downregulation of *CfabaA*. *AbaA* is a core regulator that controls asexual spore production and infection morphogenesis in fungi. Abnormal or absent expression of *abaA* leads to spore production defects and loss of pathogenicity in both *Beauveria bassiana* and *Beauveria longifolia* [42]. It is therefore plausible that L33a-induced downregulation of *CfabaA* disrupts the developmental program of *C. fimbriata*, impairing spore production and reinfection capacity. Taken together, these results suggest that L33a inhibits *C. fimbriata* through multiple mechanisms, possibly involving the suppression of *CfabaA*-mediated development and the disruption of cell wall/membrane integrity. Direct genetic evidence, such as targeted gene deletion, is needed to confirm the role of *CfabaA* in pathogenicity.

To investigate the sources of the main antifungal components of L33a, we conducted CFS and VOCs experiments (Figure 3 and Figure S1). It was found that both had antifungal effects. It is notable that the VOCs produced by L33a have a much stronger inhibitory effect on the black spot pathogen than the cell-free supernatant, indicating that VOCs dominate the antagonistic effect of L33a. Fungal spore morphology plays a significant role in responding to various stresses and is key to the development of the pathogen to new habitats. Further research found that VOCs mainly inhibit mycelial growth by suppressing spore germination. This is consistent with previous studies on Bacillus inhibiting *C. fimbriata*. *Bacillus amyloliquefaciens* SFB-1 completely inhibited spore germination at a concentration of 10^8^ CFU/mL, while transcriptome analysis showed significant changes in several genes associated with spore germination [43]. Gao et al. (2025) found that at high concentrations, *Bacillus licheniformis* BL06 could completely inhibit spore production and spore germination of the black spot pathogen [44]. Further studies revealed that the VOCs produced by L33a contained multiple antifungal components (Figure 7 and Figure S3), among which octanoic acid and PEA had an extremely excellent antifungal effect of 100%. PEA, an aromatic alcohol with a rose-like scent, is widely used in cosmetics and industrial food [45,46]. For safety reasons, we further selected PEA for spore fumigation experiments and found that the inhibitory effect on mycelial growth was consistent with the inhibitory effect of VOCs fumigation mentioned earlier, suggesting that PEA is very likely to be the main inhibitory component in VOCs. This is consistent with existing studies. The VOCs produced by *Pseudomonas chlororaphis* SPS-41 contain phenylethanol, which, together with 3-methyl-1-butanol and 2-methyl-1-butanol, can strongly inhibit the sweet potato black spot bacteria in both in vitro and in planta experiments [47]. PEA produced by *Serratia liquefaciens* JS-239 effectively inhibits mycelial growth and spore germination of *Botrytis cinerea* [48]. Inhibition of spore germination can effectively block the initial infection of the disease, and this mode of action is of great significance for postharvest disease control.

To evaluate the antagonistic effect of L33a against postharvest sweet potato *C. fimbriata*, the efficacy of L33a liquid and its VOCs against postharvest sweet potato black spot disease was tested (Figure 4 and Figure 5). In planta experiments confirmed that both L33a suspension and its VOCs could significantly reduce the occurrence of sweet potato black spot disease. This is consistent with previous studies on the inhibition of various plant diseases by *Bacillus velesiensis* [49,50]. SA and JA are core pathways for plants to defend against necrotrophic pathogens, while ABA plays a regulatory role in early stress [51,52,53]. In this study, L33a treatment significantly affected the expression of key genes in these three pathways in sweet potatoes. In the early stage of infection (6–24 h), ABA pathway genes *IbABF1*, *IbSnRK2*, and *IbAO* were rapidly upregulated, indicating that the pathogen activated ABA signaling. Biocontrol bacteria treatment significantly inhibited the overactivation of these three genes, indicating that biocontrol bacteria can reduce stress damage by negatively regulating the ABA pathway, avoid excessive energy consumption by the host, and create conditions for subsequent defense. In the middle and late stages of infection (24–96 h), the JA pathway genes *IbPDF1.2*, *IbJAZ10*, *IbOPR3,* and the SA pathway genes *IbNPR1*, *IbDML3* were continuously upregulated, and the expression levels in the L33a treatment group were higher than those in the individual pathogen group, indicating that the biocontrol bacteria cooperatively activated the JA and SA pathways. *IbOPR3* is involved in JA synthesis [54], *IbPDF1.2* is a JA-responsive defenin gene [55], and *IbNPR1* is the main regulator of SA signaling [56], and its upregulation can activate downstream *PR* genes to enhance resistance. *IbJAZ10*, as a negative regulator of JA, is upregulated and can precisely control defense intensity through negative feedback mechanisms. Meanwhile, the expression of defense enzyme genes *IbPAL*, *IbCAT*, and *IbSOD* after L33a treatment was consistent with the JA/SA pathway trend, all showing higher expression levels in the middle and late stages of the biocontrol group, indicating that biocontrol not only activates the signaling pathway but also induces downstream defense metabolism and antioxidant capacity. Taken together, these results indicate that L33a treatment is associated with early suppression of ABA-responsive gene over-induction and subsequent coordinated activation of the JA/SA pathways and defense enzyme genes, which may contribute to enhanced systemic resistance. However, these interpretations are based on transcriptional correlations; functional validation using genetic or pharmacological approaches is required to establish causal relationships between the identified genes and disease resistance.

This study has several limitations that should be considered. First, the proposed mechanism by which L33a suppresses *C. fimbriata* through downregulation of *CfabaA* and disruption of cell membrane integrity is inferred from gene expression and morphological observations; direct genetic evidence, such as targeted deletion of *CfabaA*, is lacking. Second, although PEA was identified as a highly effective VOC component, its exact contribution to total volatile activity and its effective concentration under storage conditions require further quantification. Third, host defense gene analysis relied solely on qPCR and thus provides correlational rather than causal evidence. Finally, all trials were performed under controlled laboratory conditions; pilot-scale or field experiments are necessary to evaluate practical applicability.

## 5. Conclusions

This study confirmed that *B. velezensis* L33a exerts an excellent biocontrol effect on sweet potato black spot disease. L33a and its VOCs demonstrated strong inhibitory effects on *C. fimbriata* both in planta and in vitro. Further analysis revealed that L33a disrupts the integrity of the pathogen’s cell membrane, induces hyphal abnormalities, and alters the expression levels of pathogenicity-related genes in *C. fimbriata*. Moreover, L33a inhibits spore germination via VOCs, thereby blocking the infection process. Concurrently, L33a activates the expression of defense-related genes in sweet potato, enhancing the host’s disease resistance. Monomolecular volatile fumigation experiments confirmed that PEA, a VOC produced by L33a, significantly inhibits spore germination. However, the role of *CfabaA* downregulation in pathogenicity awaits direct genetic validation, and the findings regarding host defense genes remain correlational. Future work should integrate targeted gene disruption, quantitative assessment of PEA and other VOCs, and pilot-scale storage trials to translate these findings into practical postharvest disease management strategies. In summary, these findings provide a novel strategy for controlling postharvest sweet potato diseases, indicating that L33a and its VOCs hold broad application potential in this context.

## Data Availability

The original contributions presented in this study are included in the article/Supplementary Material. Further inquiries can be directed to the corresponding authors.

## Supplementary Materials

The following supporting information can be downloaded at: https://www.mdpi.com/article/10.3390/jof12070492/s1, Figure S1: Inhibitory effect of CFS of strain L33a on *C*. *fimbriata*; Figure S2: Changes in enzyme activity-related genes in sweet potatoes infected with *C*. *fimbriata* after treatment with L33a. Figure S3: Spore germination status of *C*. *fimbriata* after PEA treatment (bar = 100 μm); Table S1: The reaction system for qPCR; Table S2: The reaction protocol for qPCR; Table S3: Primers sequence for qPCR.
