# Supplementary material for: Biocontrol Efficacy and Mechanism of Action of Bacillus velezensis L33a Against Postharvest Sweet Potato Black Rot
Source: J Fungi (Basel). 2026 Jul 3;12(7):492. doi: 10.3390/jof12070492 (PMC13412296; doi:10.3390/jof12070492)
Supplement: Supplementary file 1 [file jof-12-00492-s001.zip › jof-4371773-supplementary.pdf]

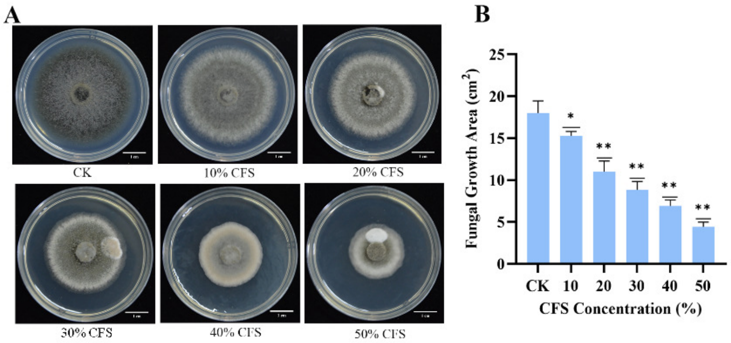

Figure S1. Inhibitory effect of CFS of strain L33a on *C. fimbriata***.** (A) Inhibitory effect of CFS on mycelial growth of *C. fimbriata*. (B) Mycelial growth area. Data are presented as mean ± standard deviation. Statistical significance was assessed using one-way ANOVA followed by Tukey's HSD test, *$p < 0.05$, **$p < 0.01$.

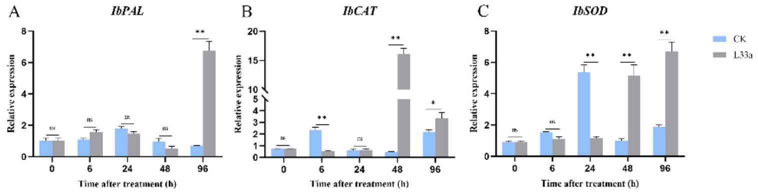

Figure S2. Changes in enzyme activity-related genes in sweet potatoes infected with *C. fimbriata* after treatment with L33a. (A) *IbPAL*. (B) *IbCAT*. (C) *IbSOD*. CK, blank control; L33a, L33a bacterial suspension (10^8 CFU/L) treatment. Values represent the mean ± SD of three independent samples, $^{**}p < 0.01$.

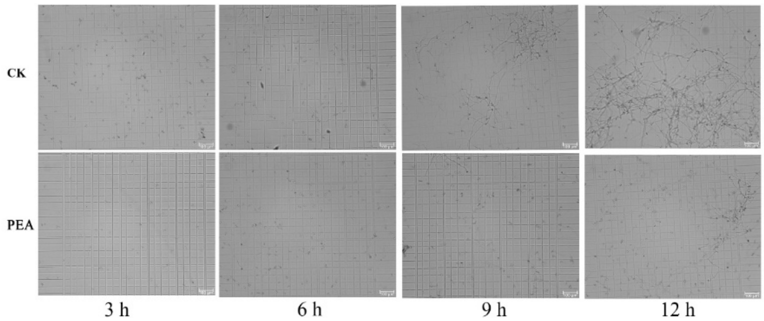

Figure S3. Spore germination status of *C. fimbriata* after PEA treatment (bar = 100 μm).

Table S1. The reaction system for qPCR.

Component	Volume (μL)
Forward primer (10 μM)	0.5
Reverse primer (10 μM)	0.5
2×SYBR Premix Ex Taq II	5
cDNA	1
ddH_2O	3

Table S2. The reaction protocol for qPCR.

Step	Temperature	Time
Initial denaturation	95 °C	30s
Denaturation	95 °C	5s
2×SYBR Premix Ex Taq II	58 °C	30s
40 cycles		
ddH_2O		
Melting curve	65 ~ 95 °C, 0.5 °C, 0.05 s	

Table S3. Primers sequence for qPCR.

Gene Name	Primer sequence (5′-3′)	Description
Ibactin	F: AGCAGCATGAAGATTAAGGTTGTAGCAC<br>R: TGGAAAATTAGAAGCACTTCCTGTGAAC	House-keeping gene
IbSOD	F: TCCTGGACCTCATGGATTTC<br>R: GCCACTATGTTTCCCAGGTC	Superoxide Dismutase
IbPAL	F: CCCTTCCCTTGGGCATTAT<br>R: AGTTGGGGATGCGGTTGAT	Phenylalanine Ammonia-Lyase
IbCAT	F: ACGCAATTCCCGGACGTGAT<br>R: AAGCCTTCCATGTGGCGGTA	Catalase
IbJAZ10	F: GCCGTTGAGCTCGATTTCTT<br>R: GCATGCTGAATATCGCGGAA	Jasmonate ZIM-domain Protein 10
IbDML3	F: CACTCTCCTTCTCCTCCGTCC<br>R: GAATGGGTTAGTTTCATCGGC	DNA Demethylase DEMETER-LIKE 3
IbOPR3	F: CGCCATCCATTCCAAGTTTAG<br>R: GGTCCCCCATAATACGCTAAC	12-Oxophytodienoate Reductase 3
IbPDF1.2	F: GGCTTCATCTCTTCGTTCATTT<br>R: GCAGTTGCTGTCCCGAGAA	Plant Defensin 1.2
IbNPR1	F: AGTCCGTTCTTCAGGAGCGT<br>R: TTCCGCAATAAAGGTAAGCC	Nonexpressor of Pathogenesis-Related Genes 1
IbPR1	F: GCAAGATTACCTAAACCCCCA<br>R: GGAGTTGGCGTAGTTCTGCG	Pathogenesis-Related Protein 1
IbAO	F: GTTACTTGCTATTCTCGGGGTG<br>R: GATGAGTCTTGGCTGGGTTG	Ascorbate Oxidase
IbSnRK2	F: GGTGCCAGTGATAACCCTCTAA<br>R: CTTGATAGCCACTTTGTGTCCAG	Sucrose Non-fermenting-1-Related Protein Kinase 2
IbABF1	F: GGAGTTTGGGACAGAAAGGAG<br>R: AAGCCCCGACAGTCACCT	ABA-Responsive Element Binding Factor 1
CfActin	F: GTCACTCACGTCGTTCCCAT<br>R: CACGCTCGGCAGTAGTAGAG	House-keeping gene
CffksA	F: ACGTTACGGTTGTAATCGCA<br>R: TTCTTGTCGTTCTTCTACGC	1,3-beta-glucan synthase component FKS1
CftcsB	F: GGACTGTACTTGTGGAACAG<br>R: GCCTTGAGCCTTCTAATAGG	Two-component system protein B
CfabaA	F: TCTGCGCCATGATTCTGTTG<br>R: GGATTCTCCAACTCCGGAGG	Conidiophore development regulator abaA
CfCTF1-BETA	F: CCACATCAGTGTGTCACCGC<br>R: CTGATGATATCTTCGGTGCT	Cutinase transcription factor 1 beta
CfCHI1	F: CCTCTGCTCCTGGCTGAGAT<br>R: AGGTCGTAGGTCATGACGTT	Chitinase 1
